# N-acetyl Cysteine Use in the Treatment of Shock Liver

**DOI:** 10.7759/cureus.7149

**Published:** 2020-02-29

**Authors:** Swetha Parvataneni, Sireesha Vemuri-Reddy

**Affiliations:** 1 Internal Medicine, Geisinger Health System, Lewistown, USA; 2 Family Medicine, Geisinger Health System, Lewistown, USA

**Keywords:** nac- n acetyl cysteine, lft - liver function tests, shock liver, severe sepsis

## Abstract

Acute liver failure is a rare, life-threatening illness accounting for about 7% of all liver-related deaths. Patients with acute liver failure are managed with supportive care initially, and if supportive care fails, liver transplantation is the definitive option for eligible candidates in liver failure. N-acetyl cysteine (NAC) has a well-established role in acetaminophen-induced liver failure and has been reported to reduce mortality in these patients. It has also been reported to provide benefit in non-acetaminophen-induced liver failure secondary to infection, drugs, and toxins. Here we report an interesting case of NAC use in an elderly patient with shock liver secondary to severe sepsis in whom liver transplantation was not an option.

## Introduction

N-acetyl cysteine (NAC) is the acetylated form of the amino acid L- cysteine. Its role in acetaminophen-induced liver failure has been well established, and the use of NAC in non-acetaminophen-induced liver failure has also been reported, although relatively few randomized trials and prospective studies have been published [1-3]. Some case reports and studies have described the use of NAC in alcoholic hepatitis, infective hepatitis, and contrast-induced nephropathy, but knowledge of its effects and relevant treatment guidelines for shock liver secondary to severe sepsis are limited. As a source of cysteine, NAC increases glutathione levels in the blood, which acts as an effective anti-oxidant by attenuating free radical-mediated tissue injury. In addition, NAC also increases levels of nitric oxide, which has vasodilatory effect that promotes increased oxygen delivery to tissues and also provides anti-inflammatory activity. NAC is available in oral as well as intravenous (IV) formulations [4]. Here we present the case report of an elderly patient with severe sepsis and shock liver secondary to severe pneumonia for whom NAC administration provided substantial improvement.

## Case presentation

**Figure 1:**
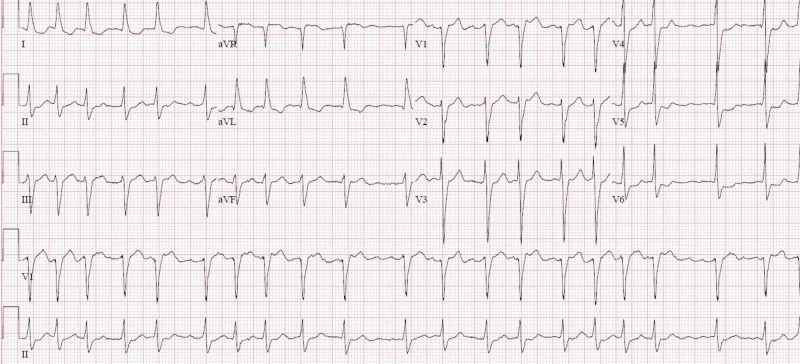
Electrocardiogram showing atrial fibrillation with rapid ventricular response

A 79-year-old male with a past medical history of atrial fibrillation and chronic lymphocytic leukemia presented to the emergency room with complaints of worsening dyspnea for two days along with cold symptoms for the previous two weeks with cough, congestion, and rhinorrhea. On examination, the patient was altered, hypotensive with systolic blood pressure (BP) of 80/40 mmHg, heart rate (HR) of 158 beats per minute, and electrocardiogram (EKG) showing atrial fibrillation with rapid ventricular response (RVR) (Figure 1).

His liver enzymes were elevated at aspartate aminotransferase (AST) 1,216, alanine aminotransferase (ALT) 736, lactic acid 9.4, and international normalized ratio (INR) = 1.93. Repeat liver function test (LFT) trended up, with AST 5,028 and ALT 2,328. He was admitted to the intensive care unit (ICU) for severe sepsis with multiorgan dysfunction, acute liver failure, and renal failure with a possible need for pressor agents. The patient was started on an IV Cardizem drip, IV fluids and a 72-hour IV NAC protocol in addition to antibiotics. His BP and lactic acidosis improved over a 48-hour period with fluids and antibiotics, and his HR improved and was well controlled. His liver enzymes trended downwards: AST 5,028 > 2,445 > 1,515 > 730 > 54 and ALT 2,328 > 2,359 > 2,278 > 1,790 > 217 over 72 hour period. His acetaminophen levels were below 5, the hepatitis panel was negative, urine drug screen was negative, and ultrasound of the abdomen and computed tomography (CT) of the abdomen and pelvis were normal. Patient has tolerated NAC without any side effects. The patient’s mental status improved over the 48-72 hour period, his vitals stabilized, and he was transferred to a step-down unit and eventually discharged on PO antibiotics with outpatient follow-up.

## Discussion

Acute liver failure is a rare, life-threatening condition characterized by sudden loss of hepatic function with liver function deterioration and abnormal coagulation with INR > 1.5 associated with encephalopathy in the absence of underlying liver disease [5]. The illness is usually less than 26 weeks in duration [6]. Approximately 2,000 individuals are affected by liver failure annually in the United States, and this disease accounts for 7% of all liver-related deaths effecting female population more than males [7]. 

The most common cause of acute liver failure is acetaminophen-induced liver failure, but other causes include viral infections, autoimmune disease, toxins, and rarely herbal and traditional medications [8-10]. Cytokine-mediated inflammatory responses from the initial injury trigger the release of free radicals and increased levels of circulatory neurotoxins and ammonia. This results in cerebral edema and multiorgan dysfunction [11-13]. The management of liver failure relies on identifying and treating the underlying cause. Various treatments such as interferon, prostaglandin, insulin and glucagon have been sought to reduce tissue injury, but they have all been proven ineffective. Many patients recover with supportive treatment, but those who fail supportive care require liver transplantation. Liver transplantation is the definitive treatment in liver failure patients and increases the survival rate from 3%-18% to 67% [14]. However, not all hospitals have access to liver transplantation centers, and many patients with co-morbidities are not good candidates for liver transplantation. Given these challenges, there will always be a need for effective medical therapy in patients with acute liver failure. NAC is well-established as a treatment for acetaminophen-induced liver failure, and recent studies have also demonstrated its efficacy in the treatment of non-acetaminophen-induced liver failure.

A prospective study reported the efficacy of oral acetyl cysteine and its impact on mortality in patients with non-acetaminophen-induced acute liver failure. The treatment group showed increased survival compared to the control group [15]. Similarly, another prospective, randomized, double-blind multi-center study determined the efficacy of IV acetyl cysteine compared to controls, reporting overall transplant-free survival rates of 40% vs 27%, respectively (P=0.043) [16]. Another randomized control study showed administration of NAC in the early stages of encephalopathy led to drastically increased transplant-free survival rates. The study concluded that this effect may be mediated by reduced levels of the cytokine IL-17 [17]. These studies are summarized in a systematic review and meta-analysis [18]. The only reported adverse events of NAC include nausea, vomiting, diarrhea or constipation, and rarely rash, fever, headache, and drowsiness.

The research on NAC in sepsis is limited, but the data described above suggest that NAC can improve encephalopathy and mortality. This is evidenced by our case where the patient presented in septic shock with multiple co-morbidities and was not a suitable candidate for liver transplantation, and thus, NAC administration has improved the survival outcomes in patients such as this.

## Conclusions

A beneficial role for NAC in non-acetaminophen-induced liver failure has been established in several randomized controlled trials and prospective studies. However, its use in severe sepsis, a life-threatening condition, has been not been thoroughly reported. This case report highlights the use of NAC in liver failure secondary to severe sepsis. Further research is needed to establish guidelines for this intervention.

## References

[1] Hu J, Zhang Q, Ren X, Sun Z, Quan Q (2015). Efficacy and safety of acetylcysteine in “non-acetaminophen” acute liver failure: a meta-analysis of prospective clinical trials. Clin Res Hepatol Gastroenterol.

[2] Mumtaz K, Azam Z, Hamid S, Abid S, Memon S, Shah HA, Jafri W (2009). Role of N-acetylcysteine in adults with non-acetaminophen-induced acute liver failure in a center without the facility of liver transplantation. Hepatol Int.

[3] Darweesh SK, Ibrahim MF, El-Tahawy MA (2017). Effect of n-acetylcysteine on mortality and liver transplantation rate in non-acetaminophen-induced acute liver failure: a multicenter study. Clin Drug Investig.

[4] Bass S, Zook N (2013). Intravenous acetylcysteine for indications other than acetaminophen overdose. Am J Health Syst Pharm.

[5] Trey C (1970). The management of fulminant hepatic failure. Progress in liver diseases.

[6] Lee WM, Squires RH Jr, Nyberg SL, Doo E, Hoofnagle JH (2008). Acute liver failure: summary of a workshop. Hepatology.

[7] Hoofnagle JH, Carithers RL, Shapiro C, Ascher N (1995). Fulminant hepatic failure: summary of a workshop. Hepatology.

[8] Björnsson E, Olsson R (2006). Suspected drug-induced liver fatalities reported to the WHO database. Dig Liver Dis.

[9] Wai CT, Tan BH, Chan CL, Sutedja DS, Lee YM, Khor C, Lim SG (2007). Drug-induced liver injury at an Asian center: a prospective study. Liver Int.

[10] Khuroo MS, Kamili S (2003). Aetiology and prognostic factors in acute liver failure in India. J Viral Hepat.

[11] Jaeschke H (2011). Reactive oxygen and mechanisms of inflammatory liver injury: present concepts. J Gastroenterol Hepatol.

[12] Nagaki M, Iwai H, Naiki T, Ohnishi H, Muto Y, Moriwaki H (2000). High levels of serum interleukin‐10 and tumor necrosis factor-α are associated with fatality in fulminant hepatitis. J Infect Dis.

[13] Bjerring PN, Eefsen M, Hansen BA, Larsen FS (2009). The brain in acute liver failure. A tortuous path from hyperammonemia to cerebral edema. Metab Brain Dis.

[14] Sales I, Dzierba AL, Smithburger PL, Rowe D, Kane-Gill SL (2013). Use of acetylcysteine for non-acetaminophen-induced acute liver failure. Ann Hepatol.

[15] Nabi T, Nabi S, Rafiq N, Shah A (2017). Role of N-acetylcysteine treatment in non-acetaminophen-induced acute liver failure: a prospective study. Saudi J Gastroenterol.

[16] Lee WM, Hynan LS, Rossaro L (2009). Intravenous n-acetylcysteine improves transplant-free survival in early stage non-acetaminophen acute liver failure. Gastroenterology.

[17] Stravitz RT, Sanyal AJ, Reisch J (2013). Effects of N-acetylcysteine on cytokines in non-acetaminophen acute liver failure: potential mechanism of improvement in transplant-free survival. Liver Int.

[18] Chughlay MF, Kramer N, Spearman CW, Werfalli M, Cohen K (2016). N-acetylcysteine for non-paracetamol drug-induced liver injury: a systematic review. Br J Clin Pharmacol.

